# Effects of Spatial Pattern Scale of Brain Activity on the Sensitivity of DOT, fMRI, EEG and MEG

**DOI:** 10.1371/journal.pone.0083299

**Published:** 2013-12-23

**Authors:** Katherine L. Perdue, Solomon Gilbert Diamond

**Affiliations:** Thayer School of Engineering at Dartmouth, Hanover, New Hampshire, United States of America; Wake Forest School of Medicine, United States of America

## Abstract

The objective of this work is to quantify how patterns of cortical activity at different spatial scales are measured by noninvasive functional neuroimaging sensors. We simulated cortical activation patterns at nine different spatial scales in a realistic head model and propagated this activity to magnetoencephalography (MEG), electroencephalography (EEG), diffuse optical tomography (DOT), and functional magnetic resonance imaging (fMRI) sensors in arrangements that are typically used in functional neuroimaging studies. We estimated contrast transfer functions (CTF), correlation distances in sensor space, and the minimum resolvable spatial scale of cortical activity for each modality. We found that CTF decreases as the spatial extent of cortical activity decreases, and that correlations between nearby sensors depend on the spatial extent of cortical activity. For cortical activity on the intermediate spatial scale of 6.7 cm^2^, the correlation distances (r>0.5) were 1.0 cm for fMRI, 2.0 cm for DOT, 12.8 for EEG, 9.5 cm for MEG magnetometers and 9.7 cm for MEG gradiometers. The resolvable spatial pattern scale was found to be 1.43 cm^2^ for MEG magnetometers, 0.88 cm^2^ for MEG gradiometers, 376 cm^2^ for EEG, 0.75 cm^2^ for DOT, and 0.072 cm^2^ for fMRI. These findings show that sensitivity to cortical activity varies substantially as a function of spatial scale within and between the different imaging modalities. This information should be taken into account when interpreting neuroimaging data and when choosing the number of nodes for network analyses in sensor space.

## Introduction

Brain activity measured by noninvasive functional brain imaging techniques is typically assumed to be generated on the cortical surface. For functional magnetic resonance imaging (fMRI), activation can be constrained to the cortical surface during reconstruction [1], [2]. Diffuse optical tomography (DOT) techniques can also constrain the reconstruction of brain activity to the cortical surface [3]. Electroencephalography (EEG) and magnetoencephalography (MEG) methods often model neural activity sources as single dipoles or a small number of patches of activity on the cortical surface [4]. The spatial extent of activity on the cortex obtained experimentally from these neuroimaging modalities and models varies widely. Data analysis methods in fMRI commonly include smoothing on the cortical surface with a gaussian kernel with a radius of 1.5–5 mm [5], [6]. Dipole models of neural activity for MEG and EEG assume that cortical activity is on an arbitrarily small spatial scale, while other methods for reconstructing MEG and EEG activity assume spatially distributed sources [7], [8].

Given these different assumptions about the spatial extent of brain activity, it can be difficult to compare the spatial resolutions of these functional imaging methods. MEG, EEG, and DOT all have spatial resolutions, as measured by localization error, on the order of a centimeter [9], [10]. The spatial resolution of fMRI is usually thought to be the same as the voxel size (usually approximately 3×3×3 mm^3^), however voxel size does not equate to the spatial scale of cortical activity discrimination because the cortical surface and brain activity does not inherently conform to the defined voxel geometries. Comparing the spatial resolution of these methods can be important when doing multimodal imaging or when comparing the results of similar studies using different imaging methods.

Different neuroimaging methods may also have differing assumptions about the nature of brain activity. In this study, we model cortical activity as occurring in multiple regions of the brain that are active simultaneously, and these regions may be of varying spatial scales. This perspective is commonly used in fMRI analysis, where recording from large numbers of voxels enables multivariate pattern analysis (MVPA) methods, which can classify the brain response to particular stimuli [11]–[13]. The question of which spatial scale is the most informative for MVPA methods is an area of active research, but the hemodynamic response on a voxel scale and larger has been shown to contain useful signals [14], [15]. Large spatial scales are also of interest in fMRI, where the cortex may be divided into active and inactive cortical regions in standard fMRI analysis. In MEG and EEG imaging, cortical activity may be modeled as localized to a single dipole at a particular timepoint. However, the spatial scale of synchronized neural activity is important when related to oscillations with local and long-range connectivity [16]. Positron emission tomography has also shown that there is widespread metabolic activity throughout the cortex even at baseline [17]. Despite the evidence for widespread, synchronized cortical activity, many studies of the spatial resolution of these functional imaging methods do not account for spatially extended cortical activity.

This study is designed to look at how the spatial scale of activity patterns on the cortical surface affect measurements in sensor space for noninvasive functional neuroimaging methods. We used simulation methods to generate spatially extended activation patterns to quantify correlations between the sensors. The spatial scale of the simulated brain activity in these patterns ranged from small, dipole-sized patches to whole hemisphere activation, as cortical activity across this broad range of spatial scales may be of interest [18]. Simulation methods are necessary for this analysis because we want to separate spatial correlations at the sensors due to extended activation regions from sensor correlations due to functional connectivity between multiple activation areas. One prior study has carried out a simpler version of this analysis for EEG by modeling the brain and head as concentric spheres [19].

Understanding how patterns of cortical activity propagate to functional imaging sensors is important for network analysis studies and multimodal imaging studies. Most network analyses using EEG, MEG, and DOT are done on the sensor level [20], [21]. Structuring network analyses in sensor space raises the important issue of determining how many nodes to include [22]. Performing our analysis in a similar way on four brain imaging methods allows for comparisons between modalities, which is especially important for multimodal imaging studies. Sensitivity to cortical activation may vary as a function of spatial scale within and between neuroimaging modalities due to the measurement biophysics. The heuristic that sensors that are located near each other are measuring similar regions of the brain may not be accurate when it comes to estimating the spatial extent of measured cortical activity.

Another area where understanding the cortical extent of activation is important is in constraining inverse problems, especially for EEG, MEG, and DOT. One approach to this problem has been to use spherical wavelets as a basis function and allow the cortical activation size to vary for MEG [7] and DOT [23]. Knowing how sensitivity inherently varies with spatial scale could be used to set appropriate weighting functions to correct for bias in measured spatial scale.

Multimodal brain imaging studies are also increasingly used as a way to understand how the same cortical activity is measured by different imaging techniques. Multimodal studies may also be designed to probe neurovascular coupling, and therefore use simultaneous neural and hemodynamic imaging methods [24]. These studies often use the heuristic that sensors that are located near each other are measuring similar regions of the brain, while not accounting for the fact that different brain imaging methods may be measuring different volumes of brain tissue due to the different measurement biophysics.

The objective of this work is to quantify how cortical activation patterns at different spatial scales propagate to functional imaging sensor measurements. We generated 1000 cortical activation patterns at 9 different spatial scales of cortical activity in a realistic head model and propagated this activity to MEG magnetometers, MEG gradiometers, EEG electrodes, DOT source-detector pairs, and fMRI voxels. This Monte Carlo experiment allows us to evaluate and compare the contrast transfer functions for the four imaging modalities. We are also able to estimate the correlation distances in sensor space and minimum resolvable spatial pattern scale. These results indicate the approximate minimum node spacing for network analysis in sensor space for each modality and the minimum spatial scale where the activation shape information can be discerned.

## Materials and Methods

### Anatomical head model

An MRI and corresponding segmentation from the BrainWeb Database [25] was selected to create the multimodal anatomical head model. The generating segmentation is voxel-based and has a probability assigned for each tissue type in each voxel. The BrainWeb segmentation contained more tissue types than we required for this study, so the “fat,” “around fat,” “muscle,” and “muscle/skin” tissues were mapped to the scalp class, and the “dura” and “bone marrow” classes were mapped to the skull class. Cortical surface models were created using Freesurfer version 4.5 [26] from the simulated T1 image. Cortical surface models included representations of the gray matter and white matter surfaces, as well as a spherical mapping of the geometry. Additional surfaces were created that mapped the midpoint between the gray and white matter boundaries. The full, high-resolution cortical geometries had over 160,000 nodes per hemisphere.

### Noninvasive functional brain imaging forward models

In general, functional brain imaging can be modeled by 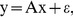
(1)where **y** is the measured signal at the sensors, **A** is the forward model, **x** is the discretized cortical activity and 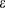 is an error term. The forward matrix **A** has the dimensions N × *M*, where N is the number of sensors for each imaging modality and *M* is the cortical source locations. In this work, the source space was the same for all modalities and consisted of 160,000 source locations on each hemisphere. The number of sensors varied according to the modality. The measurement vector **y** is a N×1 column vector, and the source vector **x** is an *M*×1 column vector. All forward models were normalized by the sensor 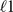 norms prior to simulating the response to different spatial scales of activity. Normalizing the forward models in this way ensures that the sensor responses with respect to spatial scale are independent of overall sensor-to-sensor gain variations within the forward models. This normalization procedure preserves sensitivity variations in the brain due to cortical folds, and the orientation, strength, and position of cortical sources.

### DOT forward modeling

**Figure 1:**
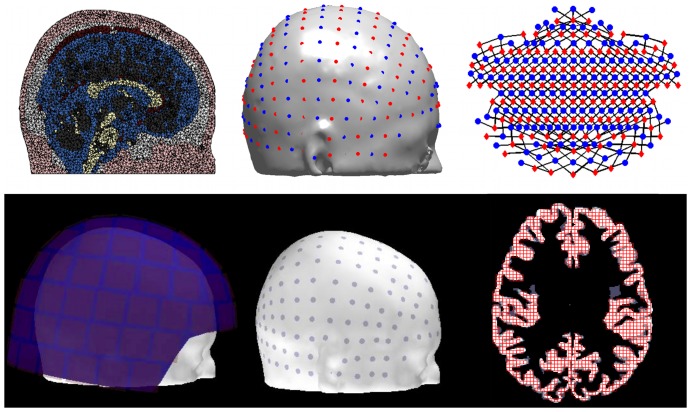
Imaging model design. Top row: Left, example slice through volumetric head mesh model. Color indicates tissue type. Center, Optode locations on the head model, red indicates sources, blue indicates detectors. Right, map with black lines showing source-detector pairs. Red diamonds are optical sources, blue circles are detectors. Bottom row: Left, MEG sensor helmet and head positioning. Center, locations of EEG electrodes on the scalp. Right, fMRI horizontal slice. Gray scale shows gray matter over three high-resolution anatomical voxels. Red grid shows the size and locations of voxels used.

DOT forward modeling was carried out as described in [27]. Briefly, an optical probe based on the 10/5 system [28] was placed on the scalp using the NFRI tools [29]. Sources and detectors were arranged in alternating rows, and the probe design included all source-detector pairs with a spacing in the range of 2–3 cm. The head model and positions of the optical sources and detectors are shown in Fig. 1. The photon propagation was modeled for each optode using MMC [30]. The forward model *A* was calculated for each source-detector pair *sd* and at each location *r* using the Rytov approximation 
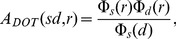
(2)where 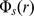 is the fluence from the source, 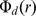 is the fluence from the detector, and the denominator is the fluence from the source as measured at the detector locaiton. The mesh-based forward model was sampled on the cortical surface to model how cortical activation propagates to the optical source-detector measurements.

### EEG and MEG forward modeling

A three-layer boundary element model was created from the segmented head model for EEG and MEG forward modeling. The segmentation used for the boundary element model was the BrainWeb probabilistic segmentation. EEG sensor locations were represented as 286 10/5 locations that were mapped to the scalp surface. The MEG sensor array used was the Neuromag Eleckta Vectorview array of 306 sensors: 102 magnetometers and 204 gradiometers. This sensor array has one magnetometer and two perpendicular gradiometers at 102 locations. The MEG sensor helmet was positioned in a realistic location using experimental data. Positions of the EEG and MEG sensors are shown in Fig. 1. The forward models for MEG and EEG, **A_MEG_** and **A_EEG_**, are represented as **A** in Eqn. 1. These models were calculated using MNE software tools [31] and the linear collocation method [32]. The forward model was calculated for the high resolution cortical node geometries using the assumption that source dipoles are perpendicular to the cortical surface. The scalp and brain compartments had a conductivity of 0.3 S/m, and the skull conductivity was 0.006 S/m.

### Functional MRI modeling

The fMRI inverse problem is well-posed assuming an adequate k-space reconstruction of the raw data, which is unlike the other functional imaging methods discussed. However, fMRI is a coarse sampling in voxels of the undulating cortical surface. For the purposes of this analysis, we devised an fMRI forward model **A_fMRI_** that averaged the cortical nodes in 3×3×3 mm^3^ voxels, represented as **A** in Eqn. 1. This forward model does not account for a spatiotemporal model of neurovascular coupling, but instead models the spatial extent of cortical activity as measured by fMRI.

Because of the high resolution of fMRI images, only one horizontal slice was chosen to show representative results. This slice was located at approximately *z* = 19 in Talairach coordinates and contained 820 simulated gray matter voxels. To be included in the analysis, voxels had to be at least 50% gray matter as calculated by a volumetric analysis, accounting for the area between the reconstructed gray and white matter surfaces. Voxels also had to include at least 4 cortical nodes. Nodes were considered to be in a voxel if their gray matter node location, white matter node location, or midpoint between the two was in the voxel. Voxels were permitted to contain cortical nodes from cortical surfaces that are on opposite banks of a sulcus. The voxels on the anatomical image are shown in Fig. 1.

### Cortical activation simulation

Cortical surface activations were simulated on 9 different spatial scales, ranging from approximately 0.03 cm^2^ to 1000 cm^2^. The number of independent nodes was increased on a roughly logarithmic level between scales (1, 6, 14, 42, 162, 642, 2565, 10242, and 40962 seed nodes per hemisphere). The independent nodes were distributed evenly over the whole cortical surface using Freesurfer's surface icosahedron downsampling capability [33] for the six smallest cortical scales. For the largest cortical scale, one node was randomly chosen as the independent node. For spatial scales with 6 and 14 independent nodes, the 6 nodes where two axes are zero in cartesian coordinates were used, for 14 independent nodes the 8 quadrant midpoint positions were also added. One thousand independently generated activation patterns were simulated at each spatial scale. The activation patterns were generated on the spherical surface and folded to respect the geometry of the cortical ribbon.

The activation patterns were constructed by generating random numbers drawn from a normal distribution at each of the independent seed nodes as located on the spherical representation of the cortical surface. Spatial smoothing was then performed in the volume by convolving the independent nodes with a spherical gaussian that had a full-width-half-max equal to the estimated spacing between independent nodes at each scale. The patterns were then *z*-transformed to ensure that the mean was still zero and the standard deviation was still one. The spherical activation pattern was then mapped to the left and right spherical cortical surfaces. Each cortical activation pattern was then randomly rotated in 3 dimensions to randomize the locations of the independent nodes and break up hemispheric symmetry. Finally, the “noncortical” locations on the Freesurfer surface representations, such as the corpus callosum, were set to zero.

The physiological meaning of positive and negative values in the activation patterns varies by imaging modality. For DOT, positive and negative activations refer to increases and decreases in chromophore concentrations, respectively. For EEG and MEG, the sign of the activation indicates the direction of the current, either into or out of the cortex. For fMRI, positive and negative cortical activations represent positive and negative blood oxygen level dependent (BOLD) signals. Despite the differences in the physiological interpretation of positive and negative activations in different imaging modalities, all modalities have established interpretations for both positive and negative signals, and positive and negative signals can cancel each other out in each modality.

**Figure 2:**
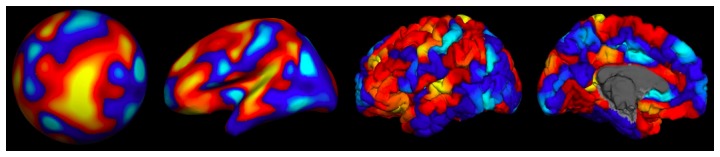
Example cortical activation pattern at 6.7^2^. Representations from left to right are spherical, inflated, pial lateral, and pial medial. Non-cortical areas are set to zero.

**Figure 3:**
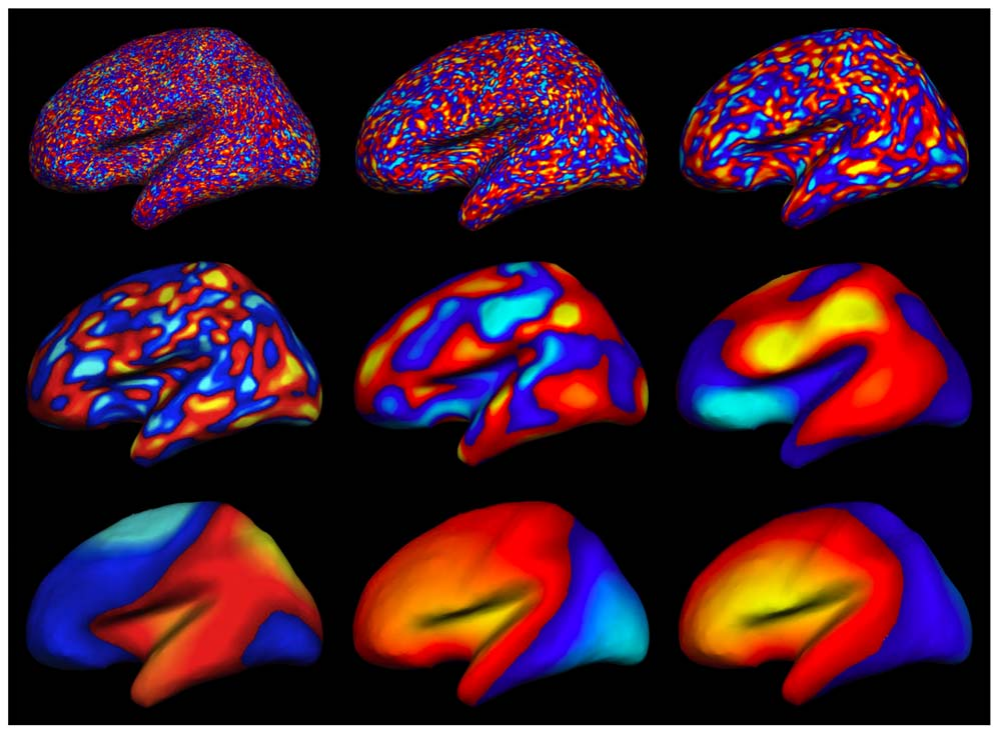
Example activation patterns shown on the inflated lateral surface. Spatial scale increases from left to right, top to bottom. The spatial scales are 0.026^2^, 0.11 cm^2^, 0.42 cm^2^, 1.7 cm^2^, 6.7 cm^2^, 26 cm^2^, 77 cm^2^, 180 cm^2^, and 1100 cm^2^.

Fig. 2 shows an example of simulated cortical activation at the 6.7 cm^2^ scale as it is generated in spherical space, on the inflated surface, and on the pial surface, which was the geometry used to propagate the activation to the sensors. We show sample activation simulations for all spatial scales displayed on the inflated cortical surface geometry in Fig. 3.

### Sensitivity, correlation distance, and resolution metrics

Sensitivity to cortical activation patterns for each modality was determined by characterizing how contrast propagates through the forward models to sensor space. The contrast transfer function (*CTF*) for each sensor at a spatial scale *s* was quantified as 
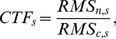
(3)where 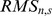 is the root-mean-square of the sensor response over the Monte Carlo instances and 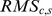 is the root-mean-square of the cortical activation. Since the cortical activations were *z*-transformed, 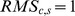. If 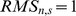, 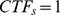 for that sensor which indicates that there was no attenuation of the cortical signal by the forward model. If 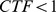, the cortical contrast has been attenuated by the forward model. The overall *CTF* for each modality is the mean of the CTF of the sensors. All forward models were normalized by 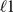 norms for each sensor prior to the *CTF* calculation.

The functional connectivity between sensors was also calculated by using a set of simulated measurements at the sensors as a pseudo-timecourse. Each sensor was used as a seed location, and then the maximum distance to a sensor correlated with *r*>0.5 was calculated using the realistic 3-dimensional sensor positions for each modality. If no sensors were significantly correlated, the minimum sensor separation was used. The mean and standard deviation over sensors was calculated for each spatial scale to represent the correlation distance for each modality.

The minimum resolvable spatial pattern scale for each modality was estimated by determining the minimum cortical activity scale where the correlation distance was one standard deviation larger than the minimum correlation distance. This quantity was calculated by fitting a smoothing spline of order 4 and smoothing parameter 0.95 to the correlation distances for each modality. This resolvable spatial pattern scale indicates the smallest scale of cortical activity where the correlations between neighboring sensors are due to the spatial extent of cortical activations and not just blurring introduced by the forward model.

## Results

### CTF for different modalities

**Figure 4:**
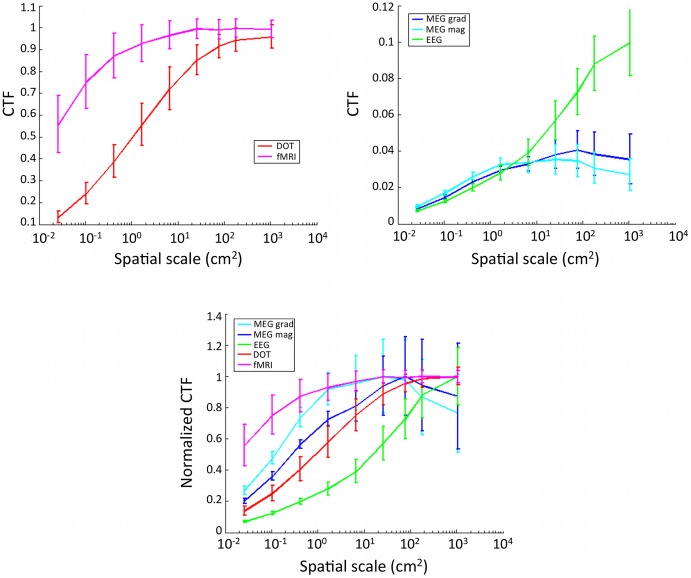
Contrast transfer functions dependency on spatial scales of brain activation. Top left: Hemodynamic functional imaging methods. Top right: Neural imaging methods. Bottom: All methods, normalized by max response.

A comparison of CTF over modality and spatial scales is shown in Fig. 4. We found that sensitivity to cortical activity generally increases with increasing spatial scale of activity. FMRI and DOT have sensitivity that stays high for spatial scales down to 26 cm^2^, though DOT falls off faster. The CTF for EEG drops off the quickest, while the CTF for MEG is less dependent on spatial scale. The MEG magnetometer and gradiometer CTF plateaus for cortical activity above the 6.7 cm^2^ scale. All other modalities have the largest CTF for the largest cortical activation scale. CTF normalized by its maximum value allows us to compare the shape of the CTF response curve between modalities. EEG falls off the fastest with decreasing spatial scale of cortical activity, while fMRI falls off the slowest, indicating the best preservation of CTF. DOT sensors and MEG magnetometers have similarly shaped CTF curves for cortical activity on the spatial scale of 77 cm^2^ and smaller.

### Sensitivity is variable over the sensor montages

**Figure 5:**
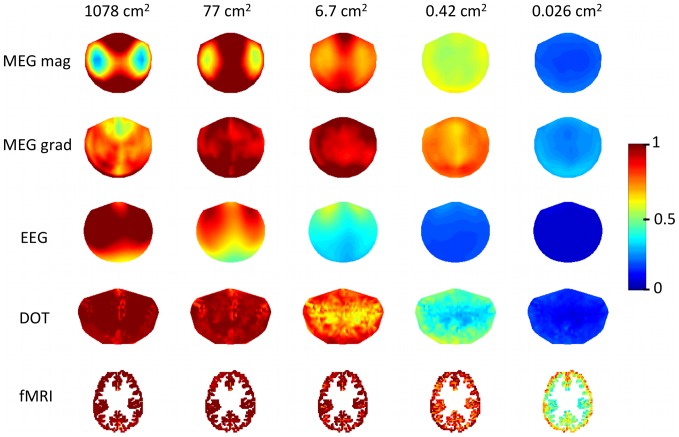
Spatial variability in CTF for five spatial scales and five sensor types. 3D sensor locations are represented in 2D. Maps are interpolated in sensor space, the top of the diagram is the most anterior sensors. Color shows normalized CTF.

A spatial map of variability in CTF for different modalities and spatial scales is shown in Fig. 5. Sensor values are interpolated over locations to better compare between modalities. Variability in CTF between sensors generally decreases with decreasing cortical spatial scale of activity, with the exception of fMRI, which has increased spatial variability with decreased activity scale. MEG magnetometers have particularly uneven sensitivity depending on location in the sensor montage for large cortical activation scales. Different spatial patterns of CTF emerge at differing spatial scales of activity, reflecting complex interactions between functional imaging sensitivity and cortical activation spatial pattern scale on the folded brain surface.

### Sensor correlations for different modalities

**Figure 6:**
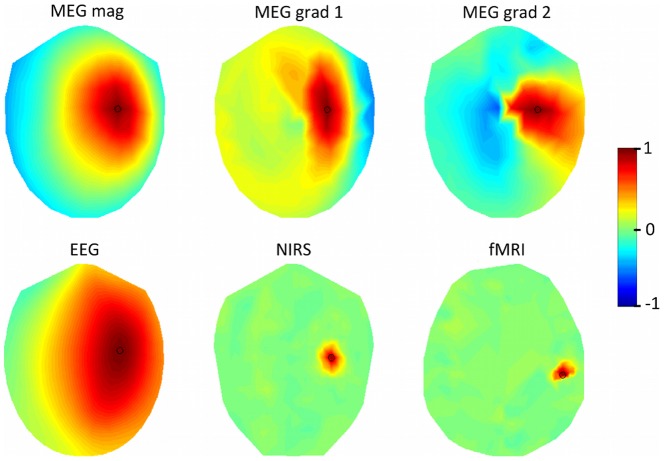
Correlations between sensors. A seed sensor is located at either exactly C4 (EEG), or near C4 (DOT, MEG), or near the central sulcus (fMRI). Cortical activity was on the scale of 1.7 cm^2^. Seed sensor is marked with a black circle.

Fig. 6 shows the correlations for one sensor and one spatial scale over all modalities. The seed sensor is exactly C4 (EEG), or near C4 (DOT, MEG), or near the central sulcus (fMRI). The simulated cortical activity was on the scale of 1.7 cm^2^. Both orientations of MEG gradiometers exhibit the effect of gradiometer direction on sensor correlations. FMRI and DOT have the smallest correlation distances. In this example, there are 16 MEG magnetometer sensors, 10 MEG gradiometer sensors, 124 EEG sensors, 2 DOT sensors, and 9 fMRI voxels with *r*>0.5 with the seed sensor for each modality.

**Figure 7:**
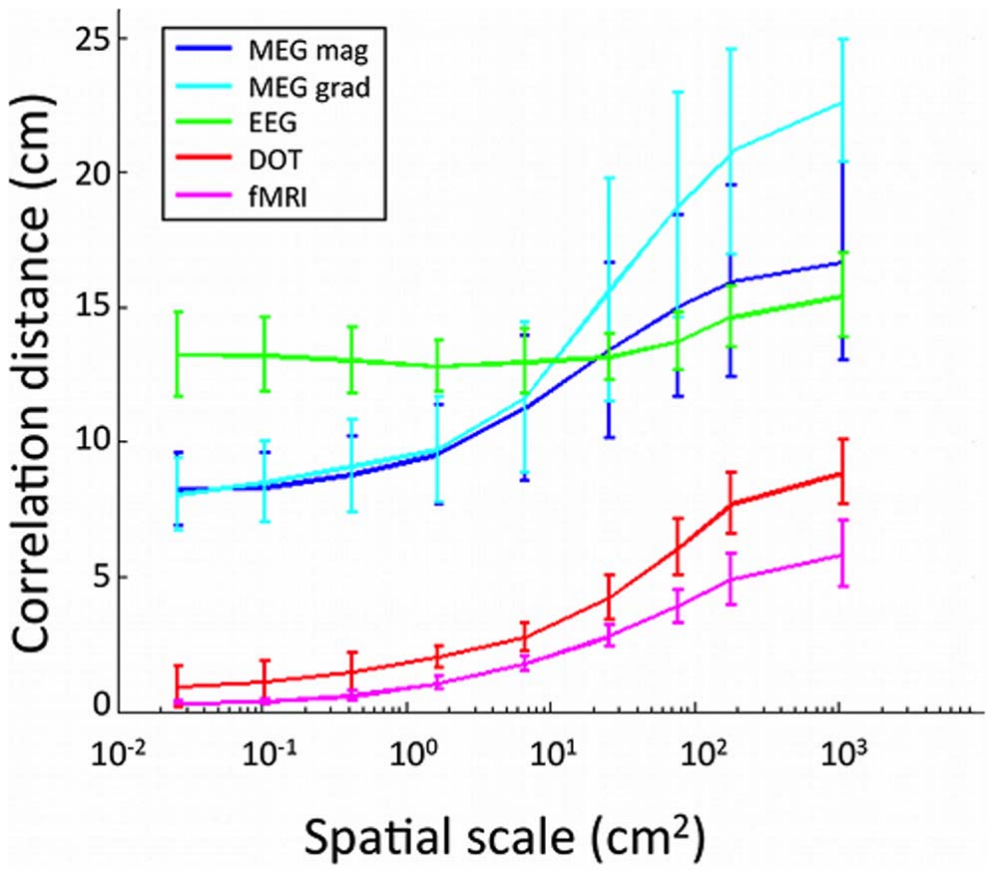
Correlation distances. Mean over sensors of the maximum distance from the seed sensor to a correlated sensor at a correlation threshold of *r*>0.5. Horizontal axis is spatial scale of modeled cortical activity.

The correlation distances for each modality and spatial scale are summarized in Fig. 7. Correlation distance generally increases with increased spatial scale of cortical activity. DOT and fMRI have shorter correlation distances for all spatial scales than MEG or EEG. EEG correlation distance remains relatively independent of spatial scale at around 13.6 cm. The resolvable spatial pattern scale was found to be 1.43 cm^2^ for MEG magnetometers, 0.88 cm^2^ for MEG gradiometers, 376 cm^2^ for EEG, 0.75 cm^2^ for DOT, and 0.072 cm^2^ for fMRI.

## Discussion

Overall, we found that sensitivity to differing spatial scales of cortical activity is highly dependent on the choice of imaging modality. DOT and fMRI appear to preserve higher spatial frequency responses, with higher CTFs and lower correlation distances than EEG and MEG. Cortical activity as measured by EEG and MEG on the surface of the scalp appears to be weighted toward spatial patterns with low spatial frequencies. The dependency of sensitivity on the extent of cortical activity suggests that node density for sensor-space network analyses of brain activity should be based on the scale of the expected cortical activation pattern. A size scale of cortical activation extent that may be of interest is at the level of cortical regions, which are typically defined as patches that are functionally related and have widths on the cortex on the order of several centimeters. One example atlas has defined cortical regions with sizes ranging from from 2 cm^2^ to 57 cm^2^, mean size of 13.7 cm^2^ and a standard deviation of 9.7 cm^2^ [34]. For cortical activations on the spatial scale of 6.7 cm^2^, we found correlation distances of 1.0 cm for fMRI, 2.0 cm for DOT, 12.8 for EEG, 9.5 cm for MEG magnetometers and 9.7 cm for MEG gradiometers. These results suggest a minimum spacing for nodes used in network analyses of brain activity using each neuroimaging modality. The correlation distance results also support the practice of ensuring that network nodes or regions of interest for EEG and MEG have sufficient spacing. Nodes may also be defined by keeping all potential sensors in the analysis while eliminating short range connections using a distance threshold [35]. For DOT, averaging over source-detector pairs to create a network node should be limited except in cases with very close spacing of the source-detector pairs (e.g. [21], [26]).

**Figure 8:**
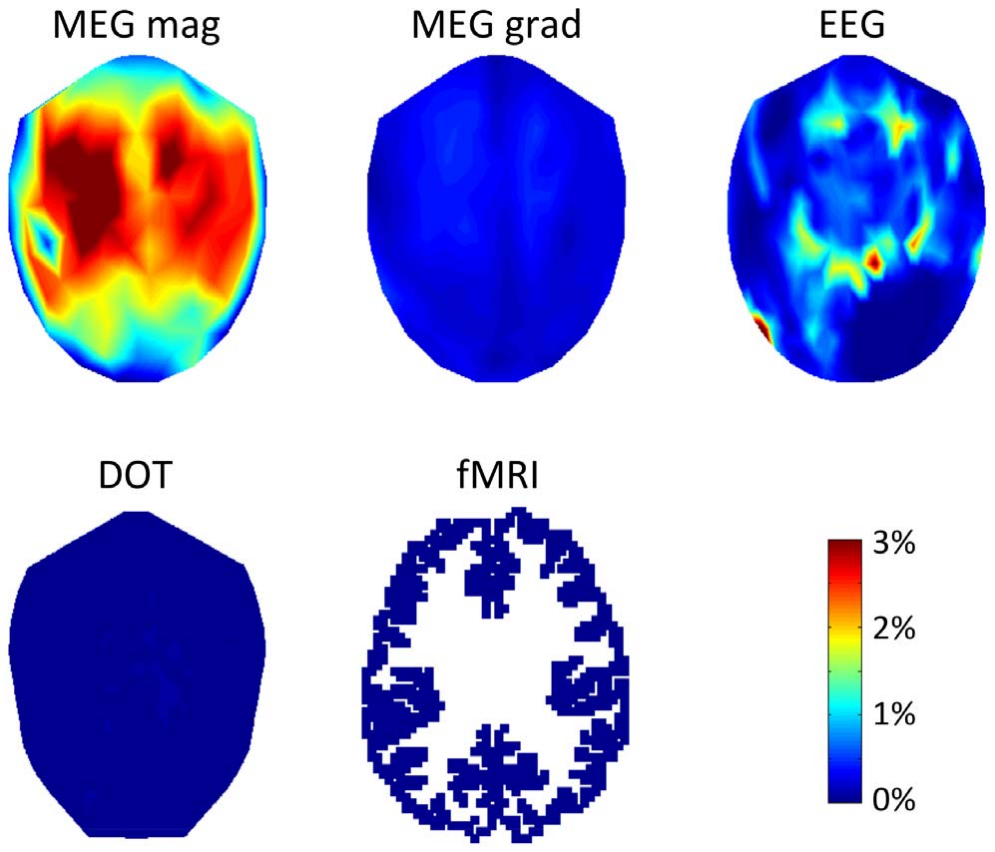
Percentage of total sources contributing to each sensor. The percentage of cortical sources where the absolute value of the forward model is at or above 50% of the maximum sensitivity value is mapped over sensor space for each modality.

The MEG magnetometers showed a relatively large amount of variability in the spatial profile of the CTF for brain activity at large spatial scales. The CTF is lower for sensors that are approximately halfway between the midline and the edge of the sensor array. This initially puzzling result has not been observed in cortical spatial resolution metrics, such as signal to noise ratio [37], source localization probability [38], or point spread functions [9], although modeling work has shown that MEG magnetometers are more sensitive to cortical orientation than EEG which may result in cancellation for extended sources [39]. However, cortical spatial resolution metrics are not directly comparable to our sensor-based metrics. Upon further investigation, it appears that the MEG sensors with low CTF for large cortical spatial scales are sensitive to an unusually large number of cortical sources due to the oblong shape of the head cerebral hemispheres. MEG magnetometers near the frontal, occipital, or midline regions of the brain are sensitive to fewer cortical source locations due to the shape of the brain and head in those locations. The percentage of sources that are greater than or equal to half of the maximum of the forward model for each sensor location are shown in Fig. 8. Sensors that are sensitive to more cortical sources will have a lower CTF when the simulated cortical patterns have a large spatial scale.

For MEG magnetometers and gradiometers, the highest sensitivity was not observed for activation patterns at the largest spatial scale. There have been recent reports of MEG cancellation for extended sources [8], [40], although these studies used smaller spatial extents for the sources and did not report what happened when there were multiple active sources. Cancellation may be more likely at larger scales of activation that may extend around the gyral and sulcal folds of the cortical surface. These large scales of activation may be seen during seizures, which are defined by widespread synchronous neural activity. Our results show that MEG and EEG are most sensitive at differing spatial scales of cortical activity, from which it follows that large cortical seizures would be easier to detect with EEG and more moderate sized activations would be easier to detect with MEG.

Resolvable spatial pattern scale was larger in EEG compared to the other modalities. Our estimated resolvable spatial scale 376 cm^2^ is on the same order of magnitude as reported elsewhere [41]. Using the Laplacian of the EEG may reduce its resolvable spatial pattern scale, perhaps up to an order of magnitude [41], which would still leave EEG as the functional imaging technique with the largest resolvable spatial pattern scale. MEG sensors have a resolvable spatial pattern scale that is similar to the resolvable spatial pattern scale of DOT, and the resolvable spatial pattern scale of DOT is an order of magnitude larger than the scale for fMRI. These results support the use of DOT and fMRI to constrain source localization for EEG [42], and combining EEG and MEG for source localization [43].

Variability in underlying physiology of the imaging modalities is not addressed by this study. We have reported different sizes of correlations at the sensors for hemodynamic and neural imaging methods based purely on the biophysics of how cortical activation extent propagates to the sensors. A complicating factor in interpreting the presented results for multimodal neuroimaging studies is that the cortical extent of the hemodynamic response may be different from the extent of the neural response. The spatiotemporal dynamics of the neurovascular coupling relationship are an area of active research [44].

The spatial resolution of fMRI has been empirically measured in the occipital cortex in response to visual stimulation. The full-width-half-max of the point spread functions of fMRI activity on the cortical surface were reported to be 3 to 4 mm at 3 tesla [45] and 2.34 mm at 7 tesla [46]. These measures correspond to a spatial extent of 0.043 to 0.13 cm^2^, and agree well with our theoretical calculation of a resolvable spatial pattern scale of 0.072 cm^2^ for fMRI.

DOT has previously been considered to be a low-resolution neuroimaging modality that is similar to EEG and MEG in reconstruction accuracy [47]. However, recent experimental work has indicated that the spatial resolution of high-density DOT is comparable to fMRI in that it is sufficient to resolve retinotopic patterns in the visual cortex [48], [49]. DOT resolution as quantified by the point-spread function is on the order of a centimeter [50], [51], a finding that is in agreement with our resolvable spatial pattern scale. Additionally, our analysis shows that sensor measurements for DOT are more local than EEG and MEG measurements, indicating that DOT may be a good choice for network analysis because it should be able to record more independent regions of the brain. High-density, whole-head DOT may be an excellent method to measure network connectivity.

EEG and MEG have a high degree of spatial blurring at the sensors according to the metrics presented in this work. The high degree of blurring is usually attributed to volume conduction [35], [52], and explains why on an absolute scale the CTF values for EEG and MEG are much smaller than those for DOT and fMRI. A similar study using dipoles instead of patches of activity found high coherence between sensors separated less that 10 cm for EEG and less than 15 cm for MEG, which is similar to the results that we found here of a correlation distance of 12.8 for EEG, 9.5 cm for MEG magnetometers and 9.7 cm for MEG gradiometers [41].

For the linear forward models used in this work, there is no effect of source strength on the input-output gain of the system response. Our patterns of cortical activation are designed to have the same distributions of source strength across the cortex, with the only change being the spatial scale of activity patches. The approach taken in the present analysis focuses attention on the effects of spatial scale but does not allow for conclusions to be drawn about the effects of source strength. Since a strong focal source can give rise to a very similar electromagnetic field as a weaker distributed source, it is possible that these two effects are not discernible in real brain activations. For example, apparently large spatial patterns measured by sensors may in fact arise from a few strong focal sources, and apparently small spatial patterns may arise from a myriad of closely located smaller active regions. When considering real data, readers should take into account that the present study focuses only on the effects of spatial scale in patterns of brain activity.

The present work brings a comparative analysis perspective on the effects of spatial pattern scale on the sensitivity of four different neuroimaging techniques. This perspective is particularly useful when evaluating experimental multimodal neuroimaging results. Neurovascular coupling studies with simultaneous measurement of neural and vascular dynamics may be carried out with EEG/fMRI [53], EEG/DOT [54], and MEG/DOT [55]. Our analysis shows that the CTF for EEG/fMRI is the least compatible pairing in terms of how contrast is propagated to the sensors at each spatial scale. One potentially good pairing of sensors would be DOT and MEG magnetometers, as they appear to have similar CTFs for cortical spatial scales that are below 77 cm^2^.

## Conclusion

We have shown that the spatial scale of cortical activity affects the sensitivity of MEG, EEG, DOT, and fMRI. We also reported a new method for defining the resolvable spatial pattern scale of functional neuroimaging modalities that accounts for the impact of extended cortical activity. We then used the method to estimate the effective resolutions of functional brain imaging modalities in terms of the resolvable scale of cortical activation patterns. These results suggest that that the nodes for network analysis performed in sensor space should account for the desired spatial scale of generating cortical activity.
